# Elective Impairment Minus Elective Disability: The Social Model of Disability and Body Integrity Identity Disorder

**DOI:** 10.1007/s11673-019-09959-5

**Published:** 2019-12-19

**Authors:** Richard B. Gibson

**Affiliations:** Centre for Social Ethics and Policy, The University of Manchester Law School, University of Manchester, Manchester, M13 9QQ UK

**Keywords:** Body integrity identity disorder, Disability theory, Impairment, Bioethics, Nonmaleficence

## Abstract

Individuals with body integrity identity disorder (BIID) seek to address a non-delusional incongruity between their body image and their physical embodiment, sometimes via the surgical amputation of healthy body parts. Opponents to the provision of therapeutic healthy-limb amputation in cases of BIID make appeals to the envisioned harms that such an intervention would cause, harms such as the creation of a lifelong physical disability where none existed before. However, this concept of harm is often based on a normative biomedical model of health and disability, a model which conflates amputation with impairment, and impairment with a disability. This article challenges the prima facie harms assumed to be inherent in limb amputation and argues in favour of a potential treatment option for those with BIID. To do this, it employs the social model of disability as a means to separate the concept of impairment and disability and thereby separate the acute and chronic harms of the practice of therapeutic healthy-limb amputation. It will then argue that provided sufficient measures are put in place to ensure that those with atypical bodily constructions are not disadvantaged, the chronic harms of elective amputation would cease to be.

In 2000, reports that surgeon Robert Smith had carried out two privately funded, therapeutic healthy-limb amputations as a means of addressing what he believed to be cases of body integrity identity disorder (BIID) drew the attention of the world’s media (Smith 2009). BIID is a non-delusional condition in which an individual feels that an aspect of their physical embodiment, most commonly one of their lower limbs, does not correspond to their self-perceived identity.^1^ Sufferers describe the presence of the body part in question making them feel “over-complete,” and thus they have a strong desire to have the limb amputated in order to address the distress and suffering its presence causes.

Unsurprisingly, the provision of “healthy” limb amputations in BIID cases draws considerable controversy, with arguments presented by opponents and proponents of the practice alike. A common argument employed by opponents to surgical interventions in BIID centres on the principle of nonmaleficence and the medical injunction of “first, do no harm.” Opponents argue that to amputate a medically healthy leg inflicts serious disproportionate harms upon that individual, not only through the amputation process itself and its associated risks but also by inflicting disability onto a previously non-disabled individual. According to this argument, using therapeutic healthy-limb amputation as a means for treating BIID breaches the principle of nonmaleficence as it invariably causes significant harms to the individual as it makes them irreversibly disabled. In response, those who tentatively support the procedure in cases of BIID argue that the harms, including the generation of disability, are potentially outweighed by the benefits, such as a significant decrease in suffering and the minimization of potential future risks (Bayne and Levy 2005; Ryan 2009; Smith 2013; White 2014).

Both sides of the nonmaleficence debate assume that amputation of a healthy limb inevitably leads to that individual becoming disabled. They merely disagree on whether the relief from suffering is worth the cost of having a disability. Both sides, therefore, purport that amputation is the single causal factor of disability, and this assumption underlies their position whether they seek to condemn or justify the practice.

This paper seeks to disrupt this underlying assumption by questioning whether a healthy-limb amputation inherently leads to disability and is, therefore, a harmful procedure per se. Can the harms commonly associated with amputation, those being the creation of a disability where none existed before, be disentangled from the physical act of removing a person’s limb? If this is the case, issues associated with nonmaleficence, as they relate to disability generation, can be evaded in the debate. This paper argues that the elective amputation of a “normal” limb does not always lead to disability.

First, it will explore, in greater detail, the employment of the principle of nonmaleficence as an argument against using healthy-limb amputation for therapeutic purposes in cases of BIID as advanced by W.J. Smith (2006), Caplan (Dotinga 2000), Müller (2007, 2009a, b), and Bruno (1997). From these arguments, it will draw the critical distinction between the potential acute harms of amputation, such as infections, thromboses, and paralysis, and the purported chronic harm which this paper is principally concerned with, that of moving from a state of non-disability to one of disability. It will also explain why the distinction between these two groups of harms matters. Second, this paper will introduce the model which it will employ as a means of separating the act of amputation, along with the imparting of impairment, from the harms commonly associated with disability, the social model of disability (SMD). This paper will then apply this separation of impairment and disability to the argument of nonmaleficence as put forward by opponents of therapeutic healthy-limb amputation. It will suggest that by employing a more complex and nuanced definition of disability, it is possible to envision the generation of impairments through the provision of healthy-limb amputation, without it necessarily creating a lifetime of disability for that individual. This paper will conclude with what such an application would mean for the ethical evaluation of therapeutic healthy-limb amputation in cases of BIID on the whole.

## Nonmaleficence and BIID

One of the central tenets within medical and clinical practices is that of *primum non nocere*, or “first, do no harm.” This obligation sits at the centre of the principle of nonmaleficence as understood by Beauchamp and Childress’ mid-level principlist theory of ethics. According to them, the principle of nonmaleficence requires that “[o]ne ought not to inflict evil or harm” (2013, 152). As such, this principle requires of us, and surgeons not only considering providing therapeutic healthy-limb amputations but all interventions, that we act with intention to refrain from causing harm to others. It is this requirement which is left unfulfilled when surgeons carry out such procedures according to opponents of the practice of healthy-limb amputation in cases of BIID. For example, W.J. Smith argued that:

…physicians are duty bound to “do no harm,” that is, they should refuse to provide harmful medical services to patients—no matter how earnestly requested. (Thus, if I were convinced that my appendix was actually a cancerous tumor, that would not justify my doctor acquiescing to my request for an appendectomy.) Finally, once the limb is gone, it is gone for good. Acceding to a request to be mutilated would amount to abandoning the patient. (Smith 2006)

The comparison W.J. Smith attempts to make between the belief that one’s appendix is cancerous and BIID in this passage is false. The belief that one’s appendix is cancerous is framed as non-factual and as such can be considered a delusion, an idiosyncratic belief maintained despite a contradiction with empirical reality. However, those with BIID recognise that the body part in question is theirs and is healthy. Their issue is that it does not correspond with their bodily image or identity, something which cannot be said to contradict empirical fact. Therefore, such a belief cannot be said to be delusional, merely unusual. However, W.J. Smith’s view on healthy-limb amputations remains clear. For him, to provide such amputations equates to a harmful medical service, a relinquishing of the patient’s best interest to that of their condition resulting in a literal surrender to the desire. Caplan also expresses similar views and argues that healthy-limb amputations would violate the Hippocratic Oath because:

The cure is not to yield to the illness and conform to the obsession. And this is not just about “do no harm.” It’s also about whether (sufferers) are competent to make a decision when they’re running around saying, “Chop my leg off.” 2 (Dotinga 2000)

For Caplan, providing healthy-limb amputations disregards other potential treatment options such as psychotherapy, cognitive behavioural therapy, or psychopharmacology, and instead harms the patient at their request.

Müller provides a similar argument against healthy-limb amputations in cases of BIID when, while discussing appeals to the principle of nonmaleficence, she argues:

According to the principle of nonmaleficence physicians must not perform amputations without a medical indication because amputations bear great risks and often have severe consequences besides the disability, for example, infections, thromboses, paralyses, necrosis, or phantom pain … Above all, an amputation causes an irreversible damage that could not be healed, even if the patient’s body image would be restored spontaneously or through a new therapy (Müller 2009b, 41).

Müller makes explicit the distinction between the potential harms associated with surgical intervention in general, which will be referred to as acute harms, and the purported harms that come as a direct result of amputation, which will be referred to as chronic harms, the generation of what Müller categorizes as a disability, one which results from “leglessness.” Differentiating between the two, particularly in the BIID/healthy-limb amputation debate, is significant because there are arguments that adequately address the concerns regarding the acute harms in amputation: first, the ambiguous nature of the principle of “first, do no harm,” second, the justification of the provision of similar surgical procedures through an appeal to a cost–benefit analysis. Pre-existence of these arguments is decisive because by addressing the acute harms associated with healthy-limb amputation via these arguments, it allows the debate to move forward and consider the chronic harm of an absentee lower limb separately.

First, though the concept of “first, do no harm” has a long tradition within the medical and health services, as argued by Ryan, the principle itself is vague and how exactly it is meant to be applied is not always clear (Ryan 2009, 27). Certainly, for the majority of surgeries to take place, healthy tissue must be damaged in order to gain access to the surgeon’s target. To employ W.J. Smith’s example of an appendectomy, an incision must be made through healthy abdominal tissue to remove the inflamed organ. This incision can, by most accounts, be considered harm, and, while the harm caused will be expected to heal, the principle of nonmaleficence is nonetheless breached as healthy tissue is intentionally damaged. However, this does not raise ethical issues because the benefits which such a surgery provides to that individual (no longer suffering from an appendicitis) are considered secondary to the practical necessities required in order to carry out such an operation. Consequently, as suggested by Sokol, it may be necessary to revise the dictum of “first, do no harm” into a more applicable and less ambiguous “first, do no *net* harm” (Sokol 2013). This would more realistically reflect the reality that while the majority of surgical and clinical interventions constitute a level of harm, or in other words maleficence, the clinician desires that the benefits conferred by such interventions outweigh those harms.

Second, for any surgery, including elective amputation, a particular group of risks are shared. Surgery can lead to unfortunate harmful complications, including infection, pneumonia, blood clotting, allergic reactions to anaesthesia, and even death. This is one reason why unnecessary surgery is often deterred. However, when performing other forms of surgery, these harms, while considered, do not prevent operations from taking place. There is the acknowledgement that despite the potential harms intrinsic to surgery, the benefits which such procedures provide outweigh the potential costs or harms. Returning to the example of an appendectomy, it carries with it the risk of wound infection, bleeding under the skin, scarring, abscesses, and even a hernia (National Health Service 2016). However, the procedure is still routinely carried out because the expected benefit outweighs these potential harms. Consequently, the surgeon expects there to be no *net* increase in harm.

This same line of reasoning can be taken concerning elective amputation in BIID cases. If there was evidence to suggest that the benefits of elective amputation were expected to provide a positive cost–benefit analysis, then the general risks associated with surgical interventions, or the acute harms of amputation, could be justified as acceptable risks and harms. Such an argument is proposed by proponents of the practice, such as Bayne and Levy (2005). Müller herself, a highly vocal critic of the practice, acknowledges this when, while discussing the principle of beneficence, she concedes that “[a]mputations could be justified according to the principle of beneficence if their benefit for the patient would override their harm” (Müller 2009b, 41). In order to demonstrate this, however, evidence would need to be provided.

The existence of evidence which demonstrates the benefits of healthy-limb amputation for those with BIID is a matter of contention in and of itself. This is because the only evidence which exists regarding the procedure’s efficacy is anecdotal (Taylor 2000; Yates 2015), individual case studies (Sorene, Heras-Palou, and Burke 2006; Berger et al. 2005; Furth and Smith 2000), or relatively small-scale empirical research (First 2005; Noll and Kasten 2014; Blom, Hennekam, and Denys 2012). What would likely be required in order to demonstrate the effectiveness of healthy-limb amputation in cases of BIID would be a large-scale, peer-reviewed, empirical study. However, such a study would be subject to a “catch-22” in that for it to gain the desired data, the surgeries would have to take place. However, the surgeries are unlikely to take place on a large enough scale until the provision of such data.

While an appeal to the arguments commonly employed to justify other forms of surgery can be used to address the acute harms of healthy-limb amputations, they are less effective at addressing the potential chronic harms caused by healthy-limb amputation. In particular, is the chronic harm which can be understood to be unique to the provision of this form of surgical intervention in cases of BIID, that being the elective creation of a disability where none existed before. To illustrate this point, let us employ a comparison to cosmetic surgery.

Cosmetic surgery is often, but not exclusively, carried out for aesthetic reasons. Procedures such as liposuction, breast enlargement, penis lengthening, and buttock lifts are all forms of elective surgery which arguably provide no therapeutic benefit, merely the adjustment of one’s physical self towards a self-idolized aesthetic standard or body construction. A construction informed by societal, economic, and other “external” influences (Nuffield Council on Bioethics 2017). It is the provision of cosmetic surgery for aesthetic reasons and the denial of healthy-limb amputation for therapeutic purposes which some proponents of the latter consider unjust. As Bayne and Levy write:

We allow individuals to mould their body to an idealized body type, even when we recognize that this body image has been formed under the pressure of non-rational considerations, such as advertising, gender-norms, and the like. If this holds for the individual seeking cosmetic surgery, what reason is there to resist a parallel line of argument for those seeking amputation? (Bayne and Levy 2005, 81)

The reason to resist, according to Patrone, is that unlike cosmetic surgery, healthy-limb amputation deliberately results in a disability. He writes:

Even if we agree that the motives of both BIID patients and of some seeking cosmetic surgery are irrational, the analogy might be thought to break down, however, when we consider the fact that, at least ideally, no disability follows from the putatively non-problematical cases of cosmetic surgery. 3 (Patrone 2009, 543)

References to disability are ubiquitous in BIID related literature. However, this paper finds Patrone’s assumption, and the assumption made by all those authors mentioned so far, that disability must necessarily follow from a healthy-limb amputation problematic. If demonstrated that disability is not a direct result of amputation, then the objection to the provision of healthy-limb amputation in cases of BIID which appeals to the chronic harms of the procedure would be addressed. To demonstrate that healthy-limb amputation does not directly cause disability, this paper employs the SMD.

## The Social Model of Disability

Biomedical models of health conceive of disability as a failure of biological functioning. According to such models “health is the absence of disease, and diseases (I include deformities and disabilities that result from trauma) *are deviations from the functional organization of a typical member of a species*” (Daniels 1985, 28, emphasis in original). Such models establish illness, deformity, and disability wholly within the individual. As such, through the utilization of science and medicine to cure or remove disease, deformity, or disability from an individual, it is possible to restore them to full health.

The SMD offers an alternative to this mechanistic understanding of the body by shifting attention from the individual to factors within their environment, including social oppression, cultural discourse, discriminative prejudices, economic influences, and physical barriers. The SMD’s goal, according to Shakespeare, is to provide “the structural analysis of disabled people’s social exclusion” (Shakespeare 2013, 214), with the final aim being the identification and altogether removal of those barriers which unjustly exclude impaired people’s participation in society. One critical method utilized by the model to achieve this, and that which differentiated it from other paradigms in disability studies during its emergence in the 1960s/70s is how it relocates disability, moving its causal factor from the individual and placing it in that person’s environment.

The SMD argues that disabilities, be they cognitive, emotional, physical, or functional, originate not as a result of individual biological “deficits.” Disability’s true origin is found in the way in which an individual’s environment is unable to accommodate their atypical needs, needs which result from that person’s impairment, such as “losing” a leg via amputation. The SMD argues that “[i]t is society which disables physically impaired people. Disability is something imposed on top of our impairments by the way we are unnecessarily isolated and excluded from full participation in society” (Union of the Physically Impaired Against Segregation and the Disability Alliance 1975). As such, disability is “the disadvantage or restriction of activity caused by a contemporary social organisation which takes little or no account of people who have physical impairments and thus excludes them from participation in the mainstream of social activities” (Union of the Physically Impaired Against Segregation and the Disability Alliance 1975). Consequently, having a leg removed through amputation would be an impairment. However, this would only cause disability if that individual exists in an environment constructed in a manner that means their impairment unjustly puts them at a disadvantage, which, unfortunately, many of us do.

What this disentangling of disability and impairment means, and what the SMD advocates, is that it is theoretically possible for a society to exist in which an individual with an impairment, such as lower limb amputation, does not experience any more difficulties in seeking out or obtaining opportunities than those without impairments. This equality in obstacle and opportunity results from an environmental construction which means that such biological differences amount to a mere-difference, much in the same manner as height, eye colour, or dietary habits. Consequently, this model offers the potential to decouple impairment and disability fully, and thereby envision the possibility of having an impairment without a disability, and vice versa, provided that the social, economic, political, and institutional environments, amongst others, are constructed in such a way to allow this to occur. 4

## The Social Model of Disability and Elective Amputations

To take a SMD view of impairment and disability as a starting point for the ethical analysis of elective amputations in cases of BIID, we are faced with a different question from the one which opponents and proponents to the practice such as Müller, Caplan, Bruno, W.J. Smith, Bayne, and Levy conceptualize and attempt to address through their indicated appeal to bodily normativity or via a favourable cost–benefit analysis. The central question concerning the principle of nonmaleficence regarding BIID has been whether it is ethically permissible to amputate a healthy leg and impart on someone a disability.

However, when employing the SMD as foundational bedrock from which to examine the discourse around the provision of healthy-limb amputation in cases of BIID, we find a different question to answer. That question being, is it ethically permissible to amputate a healthy leg and impart on someone an impairment? This change in question originates from the decoupling of disability and impairment, and the possibility of having one without the other. To amputate a leg is still to emphatically create an impairment, as understood according to the SMD. However, according to the model, this is neither sufficient nor necessary for disability to occur.

This formation of a new question, however, does not overwrite the one classically conceptualized in the nonmaleficence debate of healthy-limb amputation. It does mean, however, that an answer is needed for both: whether it is ethically permissible to create an impairment, and whether it is ethically permissible to create a disability, both in cases where none existed previously and have been elected. It is to these two questions that this paper now turns, starting with the question regarding the creation of disability.

## The Creation of Disability in Cases of BIID

As discussed, the aim of the SMD is the removal of those factors that cause disability. Consequentially, an SMD approach to the question of whether the generation of disability is permissible would garner the same answer as opponents to the provision of healthy-limb amputations like Caplan, W.J. Smith and Müller already give. They would argue that the generation of disability where none existed before does indeed breach the principle of nonmaleficence because disability, according to both groups, is a harm. However, where these groups diverge, as a result of their differing definitions of disability, is in their methods for the prevention and eventual elimination of disability.

Opponents to healthy-limb amputation in BIID cases who appeal to the principle of nonmaleficence often indicate an allegiance towards biomedical models of disability. This is characterized by Sullivan who, when discussing Bruno’s views on BIID, writes:

Disability, in Bruno’s schema is, then, the antithesis of able-bodiedness (as a natural developmental state) rather than its complement. Disability is unnatural insofar as it is the result of an accident (whether congenital or social): It is, by definition, both an aberration and an abomination and as such, is literally undesirable (Sullivan 2014, 584).

Those who oppose healthy-limb amputation utilizing the principle of nonmaleficence often show a similar preference for addressing the identity incongruity those with BIID face via an alteration of identity, rather than of the physical embodiment. Amputation gives someone “an aberration and an abomination.” As such, opponents appear to wish to preserve what they consider a complete and healthy body and thereby avoid the creation of disability.

However, according to the SMD, such a biomedical approach may impact the surface-level, “symptomatic” expression of disability, but not its source. Environmental factors would still create and reinforce disabilities that discriminate against those with atypical constructions. The way to address the creation and persistence of disability, according to the SMD, is through societal adaptation, not biological uniformity. Such adaptation would result in those of all bodily constructions being able to access available opportunities, regardless of impairment, elective or otherwise. This goal is, of course, subject to real-world limitations, complications, and resistance when it comes to the ability and willingness of individuals and societies to adapt to accommodate better those with atypical constructions, with some impairments requiring more alteration than others. However, this paper would contend that the degree of alteration required should not be a foundation for an exclusionary argument for those with more complex impairments, but rather a call for more investment and considered planning in how these individuals can be better brought into the mainstay of society.

## The Creation of Impairment in cases of BIID

Barnes’s attempts to address whether it is acceptable to create an impairment where none existed before.^5^ Barnes defends the “mere-difference theory of disability” against causation-based objections, such as making it permissible to “inflict” an impairment on an otherwise unimpaired individual. During her rebuttal, Barnes utilizes three key categories through which one can cause impairments and upon which she centres her defence. Taking Barnes’s lead, this paper will use the first of these three categories to answer the question before it, specifically in regards to BIID, this category being the causing of a non-impaired person to become impaired.^6^

Firstly then, to facilitate the discussion around the ethical considerations at play when an autonomous adult causes another autonomous adult to become impaired, Barnes uses the following example:

Amy and her nondisabled friend Ben work in a lab. After hours one day, they are playing around with lasers. Ben is not wearing any protective eyewear, and Amy knows that if she directs the laser beam at his eyes he is at risk of permanent vision loss. Nevertheless, Amy does not take any precautions to avoid directing the beam at Ben’s eyes. Ben becomes permanently blind. When Ben confronts Amy angrily about what she has done, Amy explains that she hasn’t done anything wrong. It’s not any worse to be disabled than to be nondisabled. So while she has made Ben a minority with respect to sight, she hasn’t made him any worse off (Barnes 2014, 95).

Barnes assumes that readers, in response to this case, would feel that Amy had done something grossly wrong when she blinds Ben. Barnes supports this initial reaction to Amy’s actions via an appeal to three arguments: the non-interference principle, the risk of such action, and the transition costs.

The non-interference principle, as understood by Barnes, requires of us that “you shouldn’t go around making substantial changes to people’s lives without their consent (even if those changes don’t, on balance, make them worse off)” (Barnes 2014, 95). By blinding Ben, Amy substantially and unjustly interferes in his life, thereby breaching the non-interference principle. This breach would occur even if the injury which Amy inflicts upon Ben were a reparable injury, or even if Amy does not injure Ben at all, but exercises non-consensual, undue influence over his life.

The risk factor of such an action refers not to the potential physical harm that such behaviour could lead to, but the risk to an Aristotelian concept of *eudaimonia*, or “flourishing.” Barnes posits that Ben may, after his blinding, adapt well to his new impairment and lead a life full of flourishing. However, there is the substantial risk that this will not be the case, and in fact that Ben will perceive his blindness as something which limits him, his choices, and his overall life satisfaction. This exposure to risk is a harm, and as Amy is not in a position to know which of these two outcomes is more likely, she acts unethically by exposing Ben to such a high-risk factor, regardless of whether he eventually flourishes in his new impaired state or not.

Finally, Barnes refers to the transition costs of moving from non-impaired to impaired, writing that:

… there’s a big difference between being disabled and becoming disabled. Many people find being disabled a rewarding and good thing. But there is an almost universal experience for those who acquire disability—variously called adaptive process or transitions costs—of great pain and difficulty associated with becoming disabled. However happy and well-adjusted a disabled person ends up, the process of becoming disabled is almost universally a difficult one (Barnes 2014, 96).

There is a strong case that those with impairments do have fulfilling lives, more so than typically presumed by the non-disabled and unimpaired (Albrecht and Devlieger 1999, Albrecht and Higgins 1978, Landfeldt et al. 2016), a phenomenon referred to as “The Disability Paradox” (Albrecht and Devlieger 1999). However, this fulfilment can be understood to not include the difficulties in the period in which a person transitions from unimpaired to impaired, and consequently disabled. This transition is often difficult as it usually requires the abandonment, or at least radical adaptation, or one’s goals and way of living (McMahan 2005). Therefore, becoming disabled is a harmful process, even if being disabled is not.

Consequently, while being impaired may theoretically be understood as a “mere-difference” and independently non-harmful, that is categorically different from the potentially harmful process of becoming disabled. The radical and necessary adaptation on one’s way of life and alteration of one’s goals as a result of circumstances beyond one’s control can be highly distressing and harmful. Referring back to Barnes’s previous point regarding “flourishing,” such non-elective changes put at significant jeopardy the securing of one’s *eudaimonia*.

While the example presented by Barnes, and used in this paper, is useful for exploring the issues around the creation of impairment where none existed before, it does not perfectly match onto cases involving those individuals with BIID. This is because an individual with BIID seeking a healthy-limb amputation is actively looking to gain an impairment. In Barnes’s example, Ben does not seek an impairment; Ben does not desire to be blind. It is something that is forced upon him by circumstances, which are the result of Amy’s actions. This means that the factors which Barnes identifies as being influential regarding the creation of impairment—the non-interference principle, the risk of such action, and the transition costs—as well as the analysis that follows them, while still relevant and employed in BIID ethical debate, can be understood in a different light. It is this alternative take on these principles that will now be examined.

### The Non-Interference Principle

Regarding the non-interference principle, which states that one should not make substantive changes to another’s life without their consent, we can refer back to the fact that those with BIID request to have an impairment. Such requests are in thematic contradiction to the example of Ben’s blinding as presented by Barnes. Ben did not wish to be blind; he became blind as a result of his and Amy’s reckless actions. Contrasting this, those with BIID would not be subjected to amputations against their will, rather the opposite. They would receive an amputation at their request.

Consequently, a surgeon providing such a surgical intervention would not be in breach of the non-interference principle in the same manner as Amy. They would not be substantially interfering in another person’s life without that person’s consent but acting in a manner to help bring that person’s desire into being. A surgeon carrying out an elective amputation would be facilitating a therapeutic intervention, not interference.

A potential counterargument and one which often appears in BIID debates are that those with BIID cannot consent to such medical procedures due to the influence which BIID exhorts over that person’s wishes and desires. Those with BIID are, according to such an argument, unable to exercise free and independent thought when it comes to that specific aspect of their own life. While they may be able to consent and exercise autonomy in the rest of their decisions, in regards to their ability to give informed consent for such substantial surgery, they lack capacity. This lack of capacity would mean that any surgeon carrying out an elective amputation would be operating on an individual without their consent to do so, and consequently, they breach the non-interference principle.

Such a lack of capacity, however, cannot be assumed. It would need to be demonstrated and that, as already mentioned, would be difficult as those with BIID do not express a delusional view, merely an unusual one.

### The Risk Factor

Regarding the risk factor of providing elective amputations to those with BIID and the concern of putting at jeopardy an individual’s potential for flourishing, this paper would argue that such surgical interventions secure and promote flourishing, rather than endanger it. As such, elective amputations in cases of BIID do not present exposure to substantial risk factors in the same manner or to the same degree as undesired impairments.

According to the evidence available, those with BIID are, in fact, more likely to flourish after an elective amputation then they did before. Both of the individuals who received amputations from Robert Smith have reported that they are delighted with the results of their procedures (Dyer 2000), as were nine participants who took part in First’s study who had secured amputations (First 2005). This increase in satisfaction, and concordantly a promotion of flourishing, was also noted by Noll and Kasten. They reported that, out of their interviewees who had achieved an amputation, not only did none regret their surgery, but also that “[m]ost of the persons report that they had suffered more from BIID than from any disadvantage in a life as a disabled person,” and “[t]here are no reports of regrets even when complications occurred” (2014, 231). Reports of similar increases in satisfaction and flourishing can also be found in the works of Taylor (2000), Blom, Henneham, and Denys (2012), Berger *et al.* (2005), Johnson, Liew, and Aziz-Zadeh (2011), and Furth and Smith (2000).

However, there is the possibility that those who receive a healthy-limb amputation may not flourish afterwards. The data currently available, which supports an increase in flourishing, could be subject to confirmation bias, and as such portray a deceptively optimistic prognosis for those individuals post–elective amputation, with only those who flourish after the procedure coming forward to report their experiences, leaving those dissatisfied underreported. This possibility, however, is not a compelling enough reason, in itself, to deny the possibility of healthy-limb amputations outright. Arguably, it means that more research into the efficacy of the procedure is needed, research that aims explicitly to draw out as wide a range of views from those who have undergone a healthy-limb amputation as possible, both confident and negative.

Utilizing the data that is currently available, a risk to “flourishing” would seem to be of more significant concern for those with BIID who are denied a healthy-limb amputation. This is because, despite several alternative therapeutic trials, no method of treatment has proven to have a long-lasting and consistent reductive effect on the desire for healthy-limb amputation in cases of BIID, except that of surgery. As summarized by one of Noll and Kasten’s interviewees:

Since about 11 months [post-operation] I’m living permanently with orthotics or wheel-chair. I live my everyday life freed from burnout and depression, meet friends without anxiety, can enjoy trivial things. I’ve got a new view of life, enriching me. In some way the feeling: I arrived (2014, 227).

For those with BIID, amputation does not present a risk to flourishing but promotes it through the alleviation of restrictive suffering.

### Transition Costs

The last category which Barnes identifies as a possible source for concern regarding the imparting of impairments on a previously non-impaired, existing individual, relates to the “cost” of transitioning. This concerns the harm and distress caused by the need to alter one’s desires and goals to incorporate one’s new state of embodiment. In Barnes’s example, Ben would need to adjust the way he lived his life and how he identifies with himself from that of a sighted person to one of a blind person. In regards to BIID, there would be a concern with how a person who had previously been “fully-mobile” would adjust to their new way of being, that of having one less biological limb. However, for those people with BIID, this movement from unimpaired to impaired once again differs to Ben’s similar transition.

While Ben may have been able to predict that a possible consequence of the actions of both himself and Amy playing with the lab’s lasers would be that he could go blind, this foresight would be, if existent, limited. It is highly unlikely that Ben has spent considerable time thinking about what it would be like to become blind. As a result, the sudden nature of his movement from sighted to being blind would come as a shock. Consequently, Ben has not been able to plan what his life would be like had he become blind; he has suddenly found himself in such an embodiment. This rapid and unforeseen aspect of becoming blind, compounded with the scale of such a transition, would cause a great deal of pain and suffering, and it would seem sensible to assume the same for those who suddenly found themselves in a post-amputation situation.

However, those with BIID do not become so without foresight. Indeed, many of those with BIID spend substantial portions of their lives living with the desire to become impaired due to the early onset of the disorder and its longevity (First 2005; Blanke et al. 2009; Kasten and Spithaler 2009; Blom, Hennekam, and Denys 2012). Consequently, these same individuals spend much of their lives considering what it would be like to live post-amputation, with it even being common for those who eventually go onto to achieve an amputation to “pretend” to be impaired for years beforehand, behaving as though they have their desired impairment through the use of assistive devices (Bayne and Levy 2005). Such foresight means that the “cost” of transition as it relates to the sudden need to alter one’s life plans and goals is considerably less, as the transition is not sudden, but considered, expected, and often planned.

## Conclusion

To claim that a healthy-limb amputation does not necessarily lead to disability, evidentially goes against the grain of collective popular wisdom. However, as this paper has argued, the linchpin upon which this wisdom rests is a definitional one. Depending on the definition of disability employed by those involved in the discussions around the provision of healthy-limb amputation in cases of BIID, the ethical viability of such a procedure can potentially radically differ.

To adhere to a biomedical model of disability and health requires that any “unacceptable” deviation from the statistical norm should be understood as an expression of disease, deformity, or disability, and as such emphatically undesirable. Anyone who desires such a body or condition necessarily suffers from a pathology which, in turn, leads them to desire the undesirable, and it is this desire which must be eliminated. In cases of BIID, this means the preservation of bodily integrity at the cost of the desired identity, a cost paid via appeals to more palatable treatments.

However, understanding disability in the same manner as those who support the SMD is to differentiate the body and the environment and locate disability in the gap between the two, or more accurately, in the way that the environment unjustly frustrates those of atypical construction. This means that structural as well as other differences in the body, termed impairments, move from a state of undesirability to, as Barnes terms them, “mere-differences.” Consequently, the question then becomes whether is it ethically acceptable to create an impairment where none existed before?

This paper argued that, given the unique and rare circumstances and factors in play in cases of BIID, the imparting of impairments on previously unimpaired individuals could be ethically permissible in the most common expression of the disorder, that being a single lower limb amputation. It did this by employing one of the examples used by Barnes to discuss a very similar question. However, her analyses centred upon an undesired impairment, which is the antithesis of those suffering from BIID.

Such an argument has a further potential impact regarding the BIID debate, especially in regards to the autonomy of those with BIID. Much of the literature concerning the ethical viability of providing healthy-limb amputations to those suffering from BIID has centred upon the principle of autonomy. As demonstrated in the referenced works of Caplan, Müller, and W.J. Smith, to desire such a radical surgical intervention to what appears to be an empirically healthy limb leads down the path of thinking that such an individual must have a psychological disorder, one that compromises their ability to make autonomous decisions regarding a specific aspect of their well-being. Patrone formalizes this way of thinking when he draws comparisons between people with BIID who seek a healthy-limb amputation and individuals suffering from anorexia nervosa to delegitimize the claims of the former by suggesting that BIID can be understood as a monothematic delusion in the same manner as the latter (Patrone 2009).

However, if the argument proposed in this paper were accepted, this would help to undermine the claim of those who seek to deny the provision of healthy-limb amputations in cases of BIID on autonomy grounds. This undermining is achieved through the interrogation of the idea of desiring the undesirable. To quote Caplan again, “… this is not just about ‘do no harm.’ It’s also about whether (sufferers) are competent to make a decision when they’re running around saying, ‘Chop my leg off’” (Dotinga 2000). However, if those with BIID seek to move from a state of “mere-difference” to another state of “mere-difference” for therapeutic reasons, as is the case in BIID, then this automatic questioning of autonomy would be brought into doubt, and the opportunity to address the suffering of those with BIID would be a step closer.

## Footnotes

1 Whilst the literature indicates that a desire for single lower-limb amputation is the most common expression of BIID, and as such the form which this paper will focus on, several authors have posited that the condition can express itself in other forms. See Giummarra et al. (2012); Blom, Hennekam, and Denys (2012); and Gutschke, Stirn, and Kasten (2017). These other forms of BIID raise their own unique questions regarding the viability and appropriateness of treatment, and as such, these fall outside of the scope of this paper.

2 Much has been written on the question of whether, as a result of having BIID, those seeking healthy-limb amputations have sufficient levels of individual autonomy required to not only comprehend their requests but also provide consent for such a procedure to go ahead. For examples, see R. Smith (2013), Müller (2009b), and Stirn, Thiel, and Oddo (2009).

3 By “non-problematic cases of cosmetic surgery,” this paper assumes Patrone is refering to surgeries that do not raise any bioethical concerns other than those commonly associated with cosmetic surgery (e.g., undue pressure to conform to an ideal standard, reinforcement of an unrealistic standard of beauty, the blurring of the boundary between medical caregiver and clinical sales representative, and so on).

4 It should be noted that while this distinction between impairment and disability is useful as a means of counteracting the nonmaleficence arguments presented by those opposed to elective healthy-limb amputations in cases of BIID, that is not to say that this distinction is itself unproblematic. The title of impairment relies on an ill-founded concept of “normal” functioning from which a deviation occurs, a concept that those with BIID can be understood as rejecting via their need for amputation. While it is beyond the scope of this paper to explore this juxtaposition fully, this conceptual distinction can still prove to be useful as a counterargument against appeals to the principle of nonmaleficence as it relates to harm in the context of BIID.

5 While the term of choice for Barnes is “disability,” she does note towards the beginning of *Valuing Disability, Causing Disability* that “If you accept a terminological distinction between ‘disability’ and ‘impairment,’ with ‘disability’ referring to the socially mediated effects of impairments, then you should reinterpret what follows as talk of causing impairments” (2014, 89). As this paper utilizes such a terminological distinction, Barnes’s work in the field of valuing disability is applicable to the theory purported in this paper.

6 The second and third categories which Barnes identifies and explores are the creation of an impaired person without transition costs and causing an impaired person to exist instead of a non-impaired person. The reason for the focus on the first category is due to its relevance to the creation of impairment in cases of BIID as the example provided by Barnes utilizes an existing individual with an already established identity and sense of self, something which the second and third categories alter or exclude.
